# M1 macrophage inhibits ferroptosis in *Pseudomonas aeruginosa*-induced kidney epithelial cell injury through the iNOS/ NO pathway without thiol

**DOI:** 10.3389/fcell.2025.1597160

**Published:** 2025-05-14

**Authors:** Peixiang Lu, Xiaojie Bai, Linfa Guo, Kuerban Tuoheti, Shanzhi Zhan, Tongzu Liu

**Affiliations:** ^1^ Department of Urology, Zhongnan Hospital of Wuhan University, Wuhan, China; ^2^ Department of Urology, The Fourth Affiliated Hospital of Xinjiang Medical University, Urumqi, China

**Keywords:** macrophage, *Pseudomonas aeruginosa*, epithelial cells, kidney, ferroptosis

## Abstract

**Instruction:**

Pseudomonas aeruginosa (PA) is one of the common pathogens of urinary tract infection. It can lead to urosepsis and renal damage. However, the mechanism by which P. aeruginosa affects epithelial cells is not clear.

**Methods:**

HK2 cells were treated with extracted PA supernatant (PA.sup). Different pathway inhibitors were added, and similar treatments were applied to HK2 cells co-cultured with macrophages. Cell viability, ferroptosis-related markers, and lipid peroxidation levels were measured.

**Results:**

We found that PA induced lipid peroxidation using its specially secreted 15-lipoxygenase (ploxA), thereby triggering ferroptosis in epithelial cells. And PA can also damage the GPx4/GSH defense system of epithelial cells. This effect is not through the proteasome pathway but through activating lysosomal chaperone-mediated autophagy (CMA) to reduce the host's GPx4 expression. Then macrophages inhibited lipid peroxidation and protected cells lacking GPx4/GSH through iNOS/NO•.

**Discussion:**

We demonstrated that NO• produced by macrophages can remotely prevent PA-induced ferroptosis of renal epithelial cells. When iNOS, which is responsible for NO• production, is pharmacologically inhibited, the antiferroptotic effect of NO• is reduced. In conclusion, our study reveals an intercellular mechanism for inhibiting ferroptosis, which may provide a new strategy for the host to combat P. aeruginosa -induced ferroptosis.

## 1 Introduction

Bacterial infection is very common in the clinical practice. Sepsis of the kidneys, as a potentially severe outcome of infection, which may finally develop into renal failure or acute kidney injury, has always been a topic of great concern in medical research. Nowadays, we have many ways to combat infections, such as the antibiotic and surgery. However, the body’s own defense mechanism remains an essential part of dealing with infections (Matta et al., 2017; de Miguel et al., 2022; Wang et al., 2022). Research has demonstrated that controlled cell death mechanisms are essential following infection by bacilli. Recently identified forms of controlled cell death, namely, pyroptosis and ferroptosis, have been implicated in worsening inflammation and causing cell death in compromised spinal cord tissues (Dixon et al., 2012; Stockwell et al., 2017).

Ferroptosis, an iron-dependent regulated form of cell death associated with various diseases, is characterized by the selective peroxidation of arachidonoyl phosphatidylethanolamine (ETE-PE) through the interaction of 15-lipoxygenase (15LOX) with phosphatidylethanolamine-binding protein 1 (PEBP1). Consequently, the 15LOX-2/PEBP1 complex is a principal initiator of ferroptotic cell death (Wu et al., 2002; Miriyala et al., 2016).

*Pseudomonas aeruginosa* (PA) is a Gram-negative bacterium. It has a large genome and versatile metabolism, allowing it to colonize a wide range of ecological niches. As an opportunistic microorganism, *P. aeruginosa* is present in various human tissues and organs and plays a vital role in many diseases, especially in susceptible populations, such as patients with hospital-acquired pneumonia, ventilator-associated pneumonia, cystitis, and nephritis (Tuon et al., 2012; Ito et al., 2021). Studies have found that *P. aeruginosa* is one of the main pathogens causing catheter-associated urinary tract infections (CAUTIs) (Yamaguchi and Yamada, 1991; Tsang et al., 1994). CAUTIs are among the most prevalent healthcare-associated conditions, accounting for approximately 36% of all healthcare-related infections. *P. aeruginosa* is responsible for approximately 10% of all CAUTIs and 16% of urinary tract infections (UTIs) in ICU patients (Clarke et al., 2020). The formation of biofilms on catheters is a widespread phenomenon among many pathogenic and opportunistic microorganisms, as bacteria typically grow by attaching to various surfaces in the form of colonies in natural environments (Donlan, 2001). *P. aeruginosa* produces and secretes a functional 15LOX, the transcription of which is upregulated hundreds of fold during biofilm formation (Vance et al., 2004). 15LOX is known to be a key molecule in the ferroptosis process, suggesting that 15-lipoxygenase secreted by *P. aeruginosa* may be a mediator of bacteria-induced host ferroptosis. *P. aeruginosa* can activate cytoplasmic phospholipase A2 during infection, leading to the expansion of intracellular and extracellular arachidonic acid (AA) pools. The mechanisms by which lipoxygenase affects host–pathogen interactions during infection and its potential contribution to the pathogenicity of *P. aeruginosa* require further investigation.

Nitric oxide (NO) is synthesized by nitric oxide synthases (NOSs) and is known as one of the smallest biologically active molecules, produced by a variety of cell types (Crane et al., 2009). NO plays a crucial role in neurotransmission, vascular function, host defense, and immune regulation. Three NOS isoforms have been identified: neuronal nitric oxide synthase (nNOS), inducible nitric oxide synthase (iNOS), and endothelial nitric oxide synthase (eNOS) (Donnelly et al., 1997). nNOS and eNOS are primarily expressed in neurons and endothelial cells, respectively, and are calcium dependent. Research indicates that iNOS is expressed in T cells, macrophages, and mature dendritic cells (mDCs), and regulates the differentiation and function of immune cells by participating in the nitration of key molecules involved in transcription or signaling pathways (Yakovlev et al., 2007; Hernandez-Cuellar et al., 2012; Mao et al., 2013). It is particularly highly expressed in M1-type macrophages and is almost not expressed in M2-type macrophages. Nitric oxide (NO•) plays a critical role in the host’s defense against pathogens within macrophages, exhibiting both bactericidal and bacteriostatic properties (Korhonen et al., 2005). It achieves this through two primary mechanisms: by directly binding to and inactivating iron-containing enzymes (Radi, 2018; Jin et al., 2023) and by reacting with the superoxide anion radical O2•− to form peroxynitrite (OONO-), a highly reactive species that targets the pathogen’s membrane lipids and proteins, especially protein thiols (Gow et al., 2002). This dual action of NO• contributes to its effectiveness in combating pathogens.

Ferroptosis, like other regulated forms of cell death, uses the cell’s own machinery to carry out the program (Jiang et al., 2021; Tang et al., 2021). However, what sets ferroptosis apart is its non-cell-autonomous nature, allowing it to propagate and affect neighboring cells. This is particularly relevant when considering the diffusible signaling properties of NO• and its capacity to shield M1 macrophages from ferroptosis.

In our experiment, we used a two-cell epithelial and macrophage co-culture system to elucidate a distinctive intercellular anti-ferroptotic mechanism involving NO•, particularly in the context of host–pathogen interactions. First, we observed that macrophages, when stimulated by PA, produce NO•. This NO• plays a crucial role in preventing phospholipid peroxidation. As a result, it protects not only the macrophages but also the neighboring epithelial cells from ferroptosis. Additionally, we discovered that PA targets the host’s glutathione peroxidase 4/ glutathione (GPx4/GSH) system, which leads to the degradation of GPx4 and promotes ferroptosis. To further investigate the role of epithelial cells in this process, we used an si-RNA-mediated knockdown (KD) approach in epithelial cells, which makes GPx4 in HK-2 cells insufficient. In our study, we demonstrate that PA can aggregate ferroptosis through 15-lipoxygenase (pLoxA), and iNOS/NO• in macrophage rescues epithelial cells from PA-induced ferroptotic cell death. Moreover, we confirmed that even when GPx4 is insufficient, iNOS/NO• protects cells against ferroptosis.

## 2 Materials and methods

### 2.1 Reagents

The reagents used in this study included DMEM/F12 (GIBCO, China), 1640 medium (GIBCO, China), fetal bovine serum (FBS; GIBCO, China), penicillin–streptomycin (GIBCO, China), ferrostatin-1 (FER-1; Medchemexpress, HY-100579), chloroquine (CQ; Medchemexpress, HY-17589A), MG132 (Medchemexpress, HY-13259), 1400W (Medchemexpress), ML351 (Medchemexpress, HY-18731), and baicalin (BAI; Medchemexpress, HY-N0197).

### 2.2 Cell culture

Normal human proximal renal tubular epithelial cells (HK2 cells) were purchased from Stem Cell Bank, Chinese Academy of Sciences, Shanghai, and cultured at 37°C and 5% CO_2_ in MEM/F12 medium containing 10% fetal calf serum and 100  Uml^−1^ penicillin–streptomycin. Macrophage (THP-1) from Stem Cell Bank, Chinese Academy of Sciences, Shanghai, China, were cultured at 37°C and 5% CO_2_ in 1640 medium containing 10% fetal calf serum and 100 U ml^−1^ penicillin–streptomycin.

### 2.3 Bacterial strains

*P. aeruginosa* used in this study was obtained from the ATCC (27853), cultured in LB medium at 37°C and 5% CO_2_.

### 2.4 PA supernatant collection

*P. aeruginosa* was grown in LB medium at 37°C, 220 rpm for 24 h and then re-inoculated in LB medium at an OD600 of 0.05 in 96-well vinyl microtiter plates. The supernatant was collected by centrifugation (2X) at 3,000 g for 8 min and then frozen before further use in cell culture experiments.

### 2.5 siRNA knockdown experiments

According to previous studies and some pre-experiments, we optimized the parameters. Dicer-substrate si-RNAs against GPx4 (H1263-siPLK-3-2) or control scrambled were used in this research. HK2 cells in six-well plates were incubated with a mixture containing 20 μM si-RNAs, 5 μL Lipo2000, and 250 μL Opti-MEM at 37°C according to the manufacturer’s instructions. The transfection mixture was removed after 12 h and replaced with MEM/F12 medium containing 10% fetal calf serum and 100  Uml^−1^ penicillin–streptomycin; then, cells were counted and seeded into other six-well plates for further tests.

### 2.6 Lipid ROS analysis using BODIPY-C11

To analyze lipid reactive oxygen species (ROS), epithelial cells were seeded in six-well plates for 48 h before treating with the PA supernatant. After 14 h of treatment, cells were incubated with 5 μM BODIPY 581/592-C11 for 25–30 min at 37°C. Cells were then trypsinized, washed with HBSS (2X), and resuspended in HBSS, followed by flow cytometric analysis using the FL1 channel on a flow cytometer. FlowJo software was used for analysis.

### 2.7 CCK-8 assay

Cell viability was assessed using the Cell Counting Kit-8 (CCK-8, Sangon Biotech, Shanghai, China), following the manufacturer’s instructions. We seeded HK-2 cells (1,000 cells/well) in 96-well plates, added 10 µL of CCK-8 solution to each well, and incubated at 37°C for 14 h in the dark. Finally, the absorbance was measured using a microplate reader at 450 nm for 2 h.

### 2.8 Co-culture experiments

Phorbol-12-myristate-13-acetate (PMA, 100 ng/mL) was added to THP-1 cells for 12 h, and then, LPS (100 ng/mL) and IFNγ (20 ng/mL) were added for 24 h; finally, THP-1 cells were transformed into M1 macrophages. Epithelial cells (0.08 × 10^6^ per well) were seeded in six-well plates for 48 h, and then, transwells (0.4μm, BIOFIL, TCS001006) containing M1 macrophages (3 × 10^5^ per well) were added. After incubation for 6 h, the co-cultured cells were treated with the PA supernatant, FER-1, and iNOS inhibitor 1400w. After the co-culture ended, the epithelial cells were collected for further use.

### 2.9 Western blot analysis

Total proteins were extracted by lysing tissues or cells with RIPA containing protease inhibitors. The proteins were then separated through SDS-PAGE electrophoresis and transferred to polyvinylidene fluoride (PVDF) membranes (Millipore, Shanghai, China). After blocking with 5% skim milk at room temperature for 2 h, the membranes were first incubated with secondary anti-GPx4 (1:2,000) and anti-GAPDH (1:2,000) overnight at 4°C and then with HRP-conjugating goat anti-rabbit (1:2,000) for 1 h at room temperature. The membranes were exposed on a Tanon-5200 ECL imager (Tanon, Shanghai, China).

### 2.10 GSH and MDA measurements

To access the GSH and malondialdehyde (MDA) levels, cells were collected and suspended in PBS and then lysed by freeze–thaw and bath sonication. The assay was performed using a GSH Assay Kit (Beyotime S0053) and an MDA Assay Kit (Beyotime S0131S), according to the manufacturer’s instructions.

### 2.11 Statistical analysis

Data in figure legends are presented as mean ± SD values from at least three experiments. Quantitative data were analyzed using ordinary one-way ANOVA in IBM SPSS version 27.0 (IBM Corp, Armonk, NY, United States), with *p* < 0.05 being considered statistically significant.

## 3 Results

### 3.1 PA can enhance ferroptosis in HK-2 cells

**FIGURE 1:**
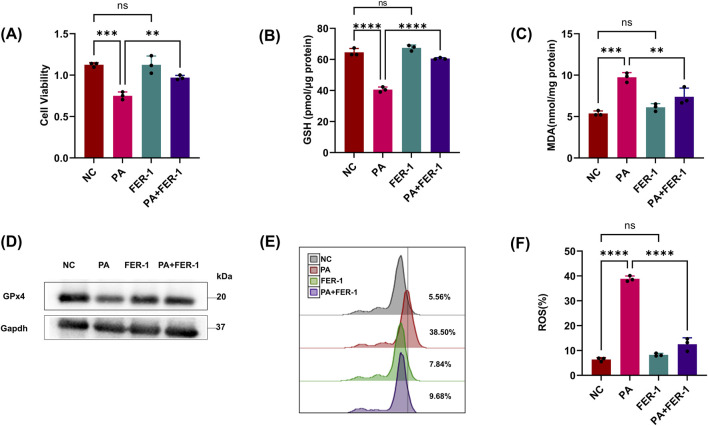
PA can trigger ferroptosis in HK-2 cells. **(A, B)** Epithelial cells were treated with the PA Sup in the presence of the indicated inhibitor; then, HK-2 cells were assessed using the CCK-8 or GSH Assay Kit. **(C)** MDA was measured in HK-2 cells after treatment with the PA supernatant or FER-1. **(D)** Western blot showing decreased expression of GPx4 in epithelial cells after treatment with the PA supernatant and rescue of GPx4 in the presence of FER-1. **(E, F)** Lipid ROS was measured in epithelial cells that were treated with or without the PA Sup and FER-1. All of the control groups (NC) consist of only HK2 cells (*p < 0.05, **p < 0.01, ***p < 0.001, and ****p < 0.0001; n = 3).

It was observed that pLoxA activity of the pathogen OMV (supernatants) and the GSH levels of host cells collectively are promising predictors of ferroptotic cell death (Dar et al., 2018). To clarify the effect of *P. aeruginosa* on renal tubular epithelial cells, we treated HK2 cells with the PA supernatant (PA sup) and found that cell viability and GSH were reduced compared to those of the control group (only HK2 cells), but this could be corrected using the ferroptosis inhibitor, FER-1 (Figures 1A,B). We further detected ferroptosis-related indicators, and the results showed that MDA and lipid ROS increased after PA sup treatment and could also be restored by FER-1 (Figures 1C,E,F). We found that treatment with the pLoxA-containing supernatant reduced the expression of GPx4 (Figure 1D). These results indicate that PA sup can promote ferroptosis in HK-2 cells.

### 3.2 PA activates the host CMA pathway for the degradation of GPx4

**FIGURE 2:**
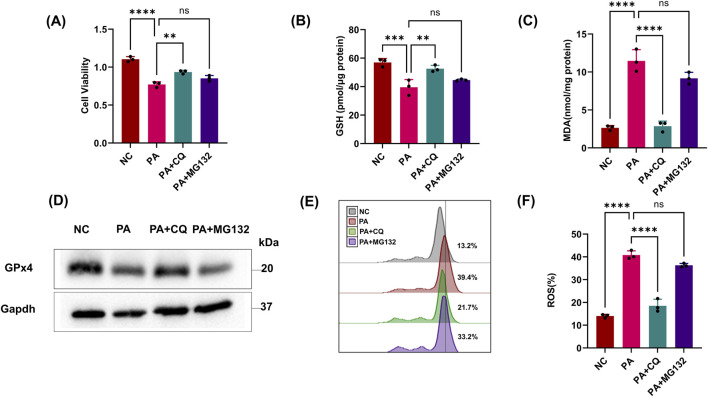
PA activates the host CMA pathway for the degradation of GPx4. **(A, B)** Epithelial cells were treated with the PA Sup in the presence of inhibitors. Then, they were assessed using the CCK-8 or GSH Assay Kit and estimated. **(C)** MDA was measured in HK-2 cells after treatment with the PA supernatant or CQ and MG132. **(D)** Western blot showing decreased expression of GPx4 in epithelial cells after treatment with the PA supernatant and rescue of GPx4 in the presence of CQ, but not in the presence of MG132. **(E, F)** Lipid ROS was measured in epithelial cells that were treated with or without the PA Sup and inhibitors. All of the control groups (NC) consist of only HK2 cells (*p < 0.05, **p < 0.01, ***p < 0.001, and ****p < 0.0001; n = 3).

The above results showed that PA inhibited the GPx4/GSH defense system of HK2 cells. As a common fact, the host protein regulatory pathways are usually one of the main targets of pathogens. So, we next explored the host protein degradation pathways targeted by PA supernatants to manipulate GPx4 protein levels. To this end, we incubated HK2 cells with PA supernatants and commonly used inhibitors of proteasome (MG132) or lysosomal (chloroquine) degradation pathways. Only CQ showed significant inhibition of reduced cell vitality and GSH, indicating that the lysosomal pathway might be involved in GPx4 degradation (Figures 2A,B). CQ also suppressed lipid ROS, whereas the proteasome inhibitor (MG132) failed to protect epithelial cells from more MDA and BODIPY oxidation (Figures 2C,E,F). Notably, CQ also rescued the reduction in GPx4 protein levels caused by PA treatment (Figure 2D). These results suggest that PA activates the chaperone-mediated autophagy (CMA) pathway in HK2 cells to degrade GPx4 and promote ferroptosis.

### 3.3 PA can aggregate ferroptosis through pLoxA

**FIGURE 3:**
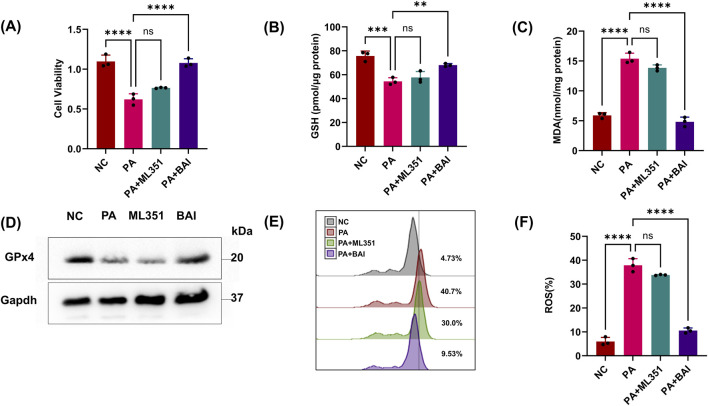
PA can aggregate ferroptosis through pLoxA. **(A, B)** Epithelial cells were treated with the PA Sup in the presence of inhibitors. Then, they were assessed using the CCK-8 or GSH Assay Kit and estimated. **(C)** MDA was measured in HK-2 cells after treatment with the PA supernatant or CQ and MG132. **(D)** Western blot showing decreased expression of GPx4 in epithelial cells after treatment with the PA supernatant and rescue of GPx4 in the presence of CQ, but not in the presence of MG132. **(E, F)** Lipid ROS was measured in epithelial cells that were treated with or without the PA Sup and inhibitors. All of the control groups (NC) consist of only HK2 cells (*p < 0.05, **p < 0.01, ***p < 0.001, and ****p < 0.0001; n = 3).

As previous studies have shown that pLoxA in PA sup can aggregate ferroptosis, we wonder whether pLoxA in PA supernatants is the main factor in HK-2 cell ferroptosis. Given the recent discovery of the leading role of human lipoxygenases (15LOX-1 and 15LOX-2) in triggering the ferroptotic cell death program, by using a specific small-molecule inhibitor of mammalian 15LOX named ML351 and a nonspecific lipoxygenase inhibitor BAI, we found that the reduction in cell viability and GSH caused by PA sup could be reversed by BAI but not by ML351 (Figures 3A,B). Similarly, ferroptosis-related indicators MDA, GPx4, and ROS LIPID detection also demonstrated that HK2 cell ferroptosis caused by PA sup could be inhibited by BAI but not by ML351 (Figures 3B–F). As there is no specific inhibitor of pLoxA, we cannot affect it directly. However, if BAI can suppress the effect of PA supernatants, but ML351, as an inhibitor of mammalian 15LOX, does not seem helpful in this experiment, it is obvious that pLoxA in the PA sup is responsible for the observed changes. All of these prove that pLoxA in PA can promote ferroptosis in HK-2 cells.

### 3.4 iNOS/NO• in macrophage rescues epithelial cells from PA-induced ferroptotic cell death

**FIGURE 4:**
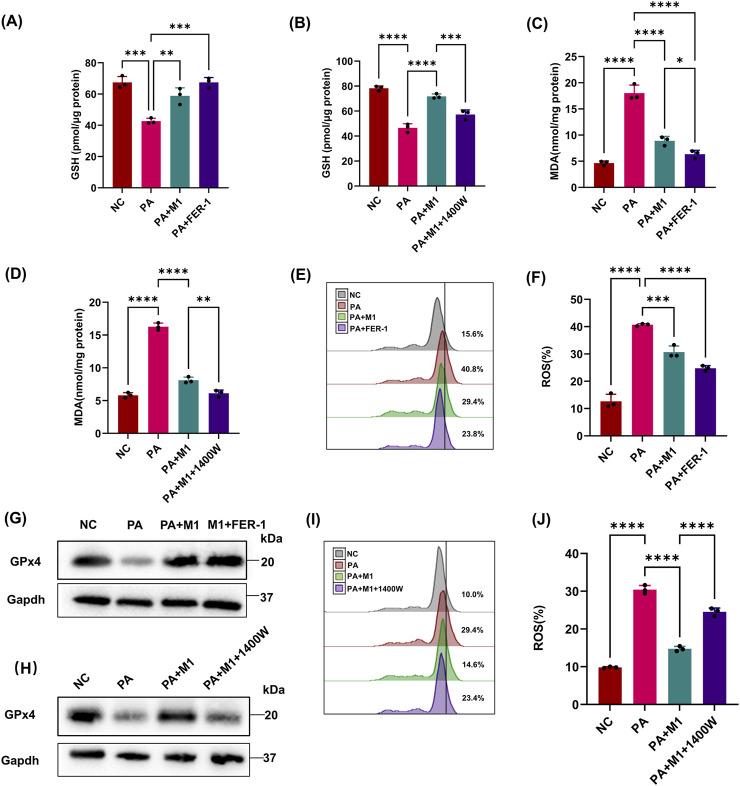
iNOS/NO• in macrophage rescues epithelial cells from PA-induced ferroptotic cell death. Epithelial cells were treated with the PA Sup and co-cultured with macrophage at the M1 state in the presence of iNOS and ferroptosis inhibitors. **(A, B)** HK-2 cells were assessed using the GSH Assay Kit and then estimated. GSH was measured after treatment with the PA supernatant or co-cultured with M1 in 1400w or FER-1. **(C, D)** MDA was measured in HK-2 cells after treatment with the PA supernatant or co-cultured with M1 in 1400w or FER-1. **(E, F, I, J)** Lipid ROS was measured in epithelial cells that were treated with or without the PA Sup and inhibitors. **(G, H)** Western blot showing decreased expression of GPx4 in epithelial cells after treatment with the PA supernatant and rescue of GPx4 in the presence of M1 and M1 plus FER-1, but not in the presence of 1400W. All of the control groups (NC) consist of only HK2 cells (*p < 0.05, **p < 0.01, ***p < 0.001, and ****p < 0.0001; n = 3).

There are many immune cells in the kidney that help epithelial cells, such as HK2 cells, and defend against microorganisms. In the context of ferroptosis, M1 macrophages are particularly important because iNOS renders them resistant to ferroptosis. Based on these studies, we explored whether the expression of iNOS in macrophages is sufficient to prevent epithelial cells from PA-induced ferroptosis. When epithelial cells and M1 macrophages were co-cultured in the presence of PA supernatants, the GSH and GPx4 levels in HK2 cells were significantly increased compared with those with PA treatment alone (Figures 4A,G), and MDA showed the diverse phenomenon (Figure 4C). As lipid peroxidation is a necessary condition for ferroptosis, we used BODIPY oxidation as an indicator of lipid peroxidation in co-culture experiments and found that M1 macrophages significantly inhibited the production of lipid peroxidation in epithelial cells (Figures 4E,F). The results indicate that M1 macrophages can alleviate PA-induced ferroptosis of HK2 cells.

Previous studies have found that M1 macrophages highly express iNOS, and iNOS/NO• can inhibit ferroptosis of epithelial cells. We wondered whether the inhibitory effect of M1 macrophages on PA-induced ferroptosis in HK2 cells was mediated by iNOS/NO•. We performed co-culture experiments in the presence of an iNOS-specific inhibitor (1400 W), which significantly abolished the protective effect of M1 macrophages on epithelial cells, making them sensitive to PA-induced ferroptosis (Figures 4B,D,H–J). These results reveal the importance of iNOS/NO• in rescuing PA-induced ferroptosis in HK2 cells by M1 macrophages.

### 3.5 iNOS/NO•-induced protection is independent of GPx4

**FIGURE 5:**
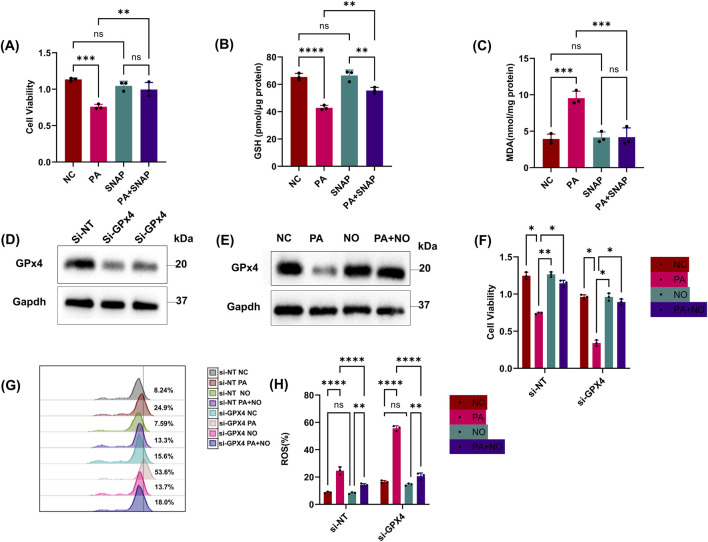
iNOS/NO•-induced protection is independent of GPx4. **(A, B)** Epithelial cells were treated with the PA Sup in the presence of inhibitors. HK-2 cells were assessed using the CCK-8 or GSH Assay Kit. **(C)** MDA was measured after treatment with the PA supernatant and SNAP. **(D)** Western blot showing decreased expression of GPx4 in epithelial cells transfected with si-RNAs against GPx4. **(E)** Western blot showing decreased expression of GPx4 after treatment with the PA supernatant and rescue of GPx4 in the presence of SNAP (NO). **(F)** Cell viability was measured in epithelial cells that were treated with or without the PA Sup and inhibitors. **(G, H)** Lipid ROS was measured in epithelial cells that were treated with or without the PA Sup and inhibitors. All of the control groups (NC) consist of only HK2 cells (*p < 0.05, **p < 0.01, ***p < 0.001, and ****p < 0.0001; n = 3).

Next, we wondered whether iNOS/NO• could protect epithelial cells from PA-stimulated ferroptosis under GPx4-restricted conditions. SNAP can increase NO in HK-2 cells as we demonstrated in experiments with conventional ferroptosis indicators (Figures 5A–C,E). We then knocked down GPx4 using si-RNA and confirmed it through cell viability and Western blotting (Figures 5D,F). We observed that reduced GPx4 protein sensitized epithelial cells to PA-induced ferroptosis (Figures 5F–H). Notably, additional NO• could still alleviate ferroptosis in GPx4-deficient cells (Figures 5G,H).

## 4 Discussion

In this research, we sought to explore the role of host-derived NO• as an intercellular defense mechanism against ferroptosis induced by PA. Our findings reveal that upon activation, NO• produced by macrophage can spread to neighboring epithelial cells, where NO• interferes with the critical factor 15LOX-2/PEBP1 complex in the ferroptotic process triggered by pathogens and shields epithelial cells from ferroptotic cell death. This unique intercellular anti-ferroptotic effect of NO• has been attributed to a variety of properties, namely, its small molecular size, lipophilicity, and high diffusion capacity (Korhonen et al., 2005). Most critically, NO• can inhibit lipid peroxidation, particularly enzymatic peroxidation catalyzed by 15LOX, including pLoxA in PA supernatants and the 15LOX-2/PEBP1 complex in mammalian cells. 15LOX-2/PEBP1 is a complex of 15LOX with a scaffold protein, PEBP1, and it catalyzes the peroxidation reaction of AA-PE and leads to the formation of the pro-ferroptotic signal 15-HpETE-PE (Korhonen et al., 2005; Dar et al., 2018).

The GPx4/GSH antioxidant system is a major defense against lipid hydroperoxide-induced ferroptosis and is increasingly recognized as a key target for disruption and manipulation by pathogens (Dar et al., 2018; Xie et al., 2023; Zheng and Guan, 2023). Failure of this system is sufficient to trigger ferroptosis. Studies have shown that *P. aeruginosa* targets multiple host pathways, including using its secreted lipoxygenases to manipulate host lipid remodeling pathways to generate pro-ferroptotic signals (Xie et al., 2023). Furthermore, *P. aeruginosa* subverts host defense mechanisms designed to neutralize these signals.

As GPx4 is a key defense mechanism against lipid hydroperoxide formation (Liu et al., 2020; Stockwell et al., 2020), its breakdown is a critical event in the process of ferroptotic cell death (Stockwell et al., 2020). Similar to known ferroptosis inducers, such as erastin, we found that treatment with the supernatant of PA activated autophagy-mediated degradation of GPx4, leading to increased production of lipid hydroperoxides and initiation of ferroptosis.

In this study, we find that treatment with PA supernatants may lead to depletion of GPx4 *via* the activation of CMA. Additionally, the application of lysosomal degradation inhibitors such as CQ resulted in reduced PA-induced lipid peroxidation and an increase in GPx4 protein levels.

NO• is a powerful signaling molecule that can act in a paracrine manner, indicating that it can influence neighboring cells and play a key role in the cardiovascular system and smooth muscle. In addition, NO• can act as an intracellular secondary messenger and play a key role in many biochemical procedures such as regulating redox homeostasis in the body (Xue et al., 2018; Cyr et al., 2020).

As the main producers during sepsis, macrophages are a major focus in immunological research and are important components of the immune system, which may develop into different forms under different immune situations. M1 macrophages typically release specific molecules, such as TNF-a, IL-6, IL-1β, and iNOS, which can exacerbate inflammation. M2 macrophages mainly release factors such as IL-10, IL-4, and TGF-β. In the immune defense, macrophages proliferate and differentiate into the M1 phenotype, in which the highly expressed iNOS generates large amounts of NO• (Chong et al., 2002). As a small molecule, NO can easily penetrate cells and act on the surroundings of the macrophages. In this way, M1 cell may play a crucial role in many diseases, such as alleviating early brain injury in experimental subarachnoid hemorrhage and reducing neuroinflammation or rheumatoid arthritis *via* the GSH/GPx4 pathway in M1 macrophages (Qu et al., 2022; Ling et al., 2024).

Our results show that iNOS expressed by M1 macrophages does not maintain the stability of GPx4 or prevent its degradation. However, it prevents the formation of lipid hydroperoxides and ferroptosis, even in the presence of deficient GPx4 levels. This finding highlights the role of iNOS/NO• in regulating ferroptosis independently of GPx4.

The properties of NO• may have important implications for tumor development or chronic infection with *P. aeruginosa*, where the local microenvironment can influence the fate of target cells (Huang et al., 2017). In chronic infections, such as those seen in cystic fibrosis patients, *P. aeruginosa* can produce NO• as part of its virulence mechanisms (Cantin et al., 2015). NO• can help the bacteria evade the host immune response and contribute to tissue damage. However, exogenous NO• can also be used therapeutically to target these bacteria as it can inhibit their growth and biofilm formation. In addition, NO• has been studied for its potentially medical role in treating neurodegenerative diseases like Alzheimer’s disease and Parkinson’s disease by protecting neurons from oxidative damage (Tewari et al., 2021), which reveals its broad usage in clinical treatment. So, it is possible that using NO•- donors may represent a promising therapeutic strategy in these patients, as well as in cases of exposure to total body irradiation after accidental or intentional catastrophic circumstances.

*P. aeruginosa*, as one of the main pathogens in CAUTIs and UTIs, particularly for ICU patients, has been under intense investigation. The use of NO•-donating compounds as a therapeutic strategy under these conditions is an area of active research (Clarke et al., 2020). Some potential advantages of NO•-based therapies include their ability to modulate multiple cellular pathways, their potential for targeted delivery, and their capacity to act as both signaling molecules and antioxidants. However, challenges remain in terms of developing safe and effective NO•-releasing agents, as well as understanding the complex interactions between NO• and other cellular components in different disease contexts.

Overall, the multifaceted roles of NO• in health and disease highlight the need for a nuanced understanding of its biology and the development of targeted therapeutic strategies that harness its beneficial effects while minimizing potential toxicities.

Lipid peroxidation is a process where ROS damage lipids, particularly the polyunsaturated fatty acids in cell membranes. This leads to the formation of end products such as MDA, 4-hydroxy-2-nonenal (HNE), and other reactive aldehydes, which can be detrimental to cellular health (Zhao et al., 2023). In the context of ferroptosis, lipid peroxidation is a central aspect as it results in the generation of phospholipid hydroperoxides (PLOOH) within cellular membranes (Dar et al., 2018).

The GPx4 system is the primary defense against ferroptosis in cells, acting as a protective mechanism by neutralizing lipid peroxides through its antioxidant activity. GPx4 is an enzyme that is crucial for the detoxification of PLOOH by converting them into their corresponding alcohols, using GSH as a reducing agent (Seibt et al., 2019; Yu et al., 2022). This process prevents the buildup of lipid peroxides, thereby safeguarding cells from ferroptosis.

Experimental evidence has demonstrated that NO has antioxidant and redox effects that are independent of the GPx4 system, ultimately inhibiting ferroptosis. In the human immune system, M1 macrophages play a crucial role and express high levels of the enzyme iNOS, which can release large amounts of NO into the immune environment (Yakovlev et al., 2007; Mao et al., 2013; Gao et al., 2022). This NO production contributes to the modulation of immune responses and has implications in the regulation of cell death pathways, including ferroptosis. Understanding the relative importance of these mechanisms can provide insights into the protective effects of NO• against ferroptosis and may have implications for the development of therapeutic strategies.

In summary, this study finds a new role for NO• as an anti-ferroptotic agent, which has significant implications in the context of host–pathogen interactions. The anti-ferroptotic properties of NO• are attributed to its small size, its ability to diffuse across cell membranes, and its capacity to react with the enzymatic machinery that generates hydroperoxy-PE and with radical intermediates of pro-ferroptotic signals. Importantly, the iNOS/NO• system operates independently of GPx4, offering protection to cells that are deficient in GPx4 against ferroptosis. Moreover, the iNOS/NO• system demonstrates great efficacy against the inducer of ferroptosis, thereby providing a universal additional layer of defense against the ferroptotic death of host epithelial cells. This discovery may lead to new therapeutic strategies to protect against ferroptosis under conditions where host–pathogen interactions play a critical role.

## Data Availability

The raw data supporting the conclusions of this article will be made available by the authors, without undue reservation.
